# The Effect of Smoking and Pre-Allogeneic Hematopoietic Cell Transplant Pulmonary Comorbidity on the Incidence of Lung Graft-Versus-Host Disease and Post-Transplant Outcomes

**DOI:** 10.3390/cancers18020295

**Published:** 2026-01-18

**Authors:** Ebaa Reda, Mohammed Kawari, Mariana Pinto Pereira, Mats Remberger, Ambrose Lau, Arjun D. Law, Rajat Kumar, Igor Novitzky-Basso, Wilson Lam, Ivan Pasic, Armin Gerbitz, Auro Viswabandya, Dennis D. Kim, Jeffrey H. Lipton, Jonas Mattsson, Fotios V. Michelis

**Affiliations:** 1 Hans Messner Allogeneic Blood and Marrow Transplant Program, Princess Margaret Cancer Centre, Toronto, ON M5G 2M9, Canada; 2 Department of Medical Sciences, Uppsala University and KFUE, Uppsala University Hospital, 751 85 Uppsala, Sweden; 3 Division of Respirology, University Health Network, Toronto, ON M5G 2C4, Canada; 4 Division of Medical Oncology and Hematology, Princess Margaret Cancer Centre, 610 University Ave., Toronto, ON M5G 2M9, Canada

**Keywords:** allogeneic hematopoietic cell transplantation, smoking, pulmonary comorbidity, retrospective, transplant outcomes, chronic graft versus host disease

## Abstract

Background/Objectives: Smoking is linked to an increased risk of pulmonary complications and adverse outcomes following allogeneic hematopoietic cell transplantation (Allo-HCT). Unfortunately, data is rarely correlated with the incidence of GVHD and does not show whether smoking has a negative impact independent from underlying pulmonary comorbidities. Methods: We retrospectively analyzed 407 patients who underwent Allo-HCT between January 2019 and May 2021 and evaluated the impact of smoking history and pre-transplant pulmonary comorbidities on the risk of outcomes including graft-versus-host disease (GVHD), overall survival (OS), and non-relapse mortality (NRM). Results: Patients were divided into the following groups: Group A: smokers with pre-transplant pulmonary comorbidity, 40 pts (9.8%); Group B: non-smokers with pre-transplant pulmonary comorbidity, 71 pts (17.4%); Group C: smokers without pre-transplant pulmonary comorbidity, 105 pts (25.8%); and Group D: non-smokers without pre-transplant pulmonary comorbidity, 191 pts (46.9%). Smokers were also grouped by their smoking history (<10 pack-years (59 pts), 11 to 25 pack-years (50 pts), and >25 pack-years (35 pts)) and by smoking recency: Recent (until Allo-HCT), Former (quit > 1 year ago), and Remote smokers (quit > 10 years ago). Our results showed that Group A demonstrated increased chronic lung GVHD compared to the other groups (*p* = 0.01). The 3-year OS was lowest in Group A at 45.0%, compared to 70.4%, 62.4%, and 69.4% (*p* = 0.006), and the NRM was highest at 37.5%, compared to 15.5%, 18.2%, and 14.7% in Groups B, C, and D, respectively (*p* = 0.001). Smoking recency and higher pack-year dose were associated with worse outcomes. Conclusions: Our study demonstrated the negative synergistic effect of smoking history and pre-transplant pulmonary comorbidities on the incidence of lung GVHD, OS, and NRM.

## Simple Summary

Smoking is linked to an increased risk of pulmonary complications and adverse outcomes following allogeneic hematopoietic cell transplantation (Allo-HCT). We retrospectively analyzed 407 patients who underwent Allo-HCT between January 2019 and May 2021 and evaluated the impact of smoking history and pre-transplant pulmonary comorbidities on post-transplant outcomes. Patients were divided into four groups: Group A: smokers with pre-transplant pulmonary comorbidity; Group B: non-smokers with pre-transplant pulmonary comorbidity; Group C: smokers without pre-transplant pulmonary comorbidity; and Group D: non-smokers without pre-transplant pulmonary comorbidity. Smokers were also grouped by their smoking history in pack-years and smoking recency. Group A showed the worst outcomes in terms of chronic lung graft-versus-host disease (GVHD), overall survival, and non-relapse mortality. Similarly, smoking recency and higher pack-years were also associated with worse outcomes. Our results showed the negative synergistic effect of smoking history and pulmonary comorbidity on the incidence of lung GVHD and survival.

## 1. Introduction

Allogeneic hematopoietic cell transplantation (Allo-HCT) is the only potentially curative treatment option for many malignant and non-malignant hematological diseases. Unfortunately, it carries with it a significant risk for a multitude of adverse effects [1]. Graft-versus-host disease (GVHD) continues to be a common complication following Allo-HCT, and remains a major cause of morbidity and mortality in transplant recipients [2]. Many studies had previously linked severe chronic GVHD with a worse prognosis [3,4]. Other studies have specifically linked pulmonary chronic GVHD with an overall higher mortality [5,6,7,8,9].

In the context of Allo-HCT, many studies have reported the negative impact of smoking on transplant outcomes, including a lower overall survival (OS), increased non-relapse mortality (NRM), and disease relapse, as demonstrated by Marks et al. [10]. There were reports of smoking also being associated with a worse OS and a longer transplant hospital stay in a paper by Ehlers et al. compared to not smoking [11], and an increased risk of pulmonary complications and pulmonary transplant-related mortality (PTRM), as shown in a review by Savani et al. [12].

Multiple other studies and reviews have provided different perspectives on the subject matter [13,14,15,16,17,18,19,20,21,22,23]. To our knowledge, however, it remains unclear whether smoking adversely affects the outcomes of Allo-HCT independently of pre-transplant pulmonary comorbidities. This study aims to investigate the impact of smoking history, total smoking dose, and pre-transplant pulmonary comorbidities, as defined by the Hematopoietic Cell Transplantation-Specific Comorbidity Index (HCT-CI), on Allo-HCT outcomes, focusing on OS, NRM, and the incidence of pulmonary GVHD.

## 2. Patients and Methods

We conducted a retrospective analysis of a sample of 407 patients undergoing Allo-HCT at the Princess Margaret Cancer Centre, Toronto, Canada, between January 2019 and May 2021. Patient data were collected through chart review and summarized through descriptive statistics. This study was approved by our institutional Research Ethics Board of the University Health Network (study ID 22-6023 authorized 27 April 2023); all patients involved had previously consented to the collection and use of their data.

We analyzed the impact of pre-transplant pulmonary comorbidity, smoking history, and total smoking dose on transplant outcomes. Pre-transplant pulmonary comorbidity was defined according to the Hematopoietic Cell Transplantation Comorbidity Index (HCT-CI) [24]: moderate pulmonary comorbidity was defined as Dlco and/or FEV1 66–80% or dyspnea on slight activity (when pulmonary function tests were not available), and severe pulmonary comorbidity was defined as Dlco and/or FEV1 ≤ 65% or dyspnea at rest or requiring oxygen.

Patient- and transplant-related characteristics are summarized in Table 1. Among our sample, the median age was 58 years (18–76). Of these patients, 94 (23.1%) received grafts from human leukocyte antigen (HLA)-matched related donors, 195 (47.9%) from matched unrelated donors, while 79 (19.4%) and 39 (9.6%) had haploidentical and HLA 9/10 mismatch unrelated donors, respectively. The majority of the transplants included in our analysis were indicated for the treatment of Acute Leukemia (myeloid and lymphoid) (n = 259, 63.6%), followed by MDS/MPN (n = 92, 22.6%), Lymphoma (n = 22, 5.4%), non-malignant indications (n = 20, 4.9%), and Chronic Leukemia (n = 14, 3.4%). Conditioning regimens included both reduced-intensity conditioning (RIC) (n = 246, 60.4%) and myeloablative conditioning (n = 161, 39.5%). Two-thirds of the patients (n = 264, 64.9%) received dual in vivo T-cell depletion for GVHD prophylaxis, consisting of antithymocyte globulin (ATG) and post-transplant cyclophosphamide (PTCy), while 81 patients (19.9%) received ATG alone, and 54 patients (13.3%) received PTCy alone (Table 1). All patients had a calcineurin inhibitor included in their GVHD prophylaxis regimen.

### 2.1. Pulmonary Comorbidity/Pre-Transplant Smoking Groups

Detailed smoking history was documented at consultation and/or pre-transplant assessment for all patients; 145 patients were determined to have a smoking history (36% of the entire cohort). Four groups were formed as such (Table 2):

• Group A: Smokers with pre-transplant pulmonary comorbidity, 40 pts (9.8%).
• Group B: Non-smokers with pre-transplant pulmonary comorbidity, 71 pts (17.4%).
• Group C: Smokers with no pre-transplant pulmonary comorbidity, 105 pts (25.8%) (one patient was missing details about their smoking history).
• Group D: Non-smokers with no pre-transplant pulmonary comorbidity, 191 pts (46.9%).

Smokers (n = 145) were also grouped based on their smoking history (Table 2): <10 pack-years (59 pts, 14.5%), 11 to 25 pack-years (50 pts, 12.3%), and >25 pack-years (35 pts, 8.6%). We also categorized smokers based on their current or previous smoking history into Recent (quit no longer than one year earlier, n = 20), Former (quit 1–10 years ago, n = 44), and Remote smokers (quit > 10 years ago, n = 81).

### 2.2. Statistical Analysis

Patient and disease characteristics were reported using descriptive statistics (Table 1). Study endpoints of interest included 3-year OS, NRM, and cumulative incidence of relapse (CIR), as well as grade III-IV acute GVHD (at 100 days following Allo-HCT) and moderate to severe chronic GVHD (within 2 years following Allo-HCT), with a focus on pulmonary chronic GVHD [25]. Additional study endpoints included the number of days of inpatient hospital and intensive care unit (ICU) stay, and viral reactivation rates of Cytomegalovirus (CMV) and Epstein–Barr Virus (EBV). Survival analysis was performed and plotted on Kaplan–Meier curves, while hospital stays were represented on bar graphs. The multivariate analysis was performed using the Cox proportional hazard regression model, with variables including age and HCT-CI and Disease Risk Index (DRI) scores [26], as well as the pre-defined pulmonary comorbidity/smoking status groups and the number of pack-years of smoking for those with a smoking history. All statistical analyses were performed using the freely available EZR 1.61 software and Statistica 14.2 (TIBCO, Palo Alto, CA, USA).

## 3. Results

### 3.1. Patient Characteristics

The distribution of patients based on their smoking history and pre-transplant pulmonary comorbidities are summarized as described above in Table 2. The exact distribution of smokers based on their pack-years in Groups A and C is further detailed in Table 2.

Further analysis of our patient population by pulmonary comorbidity/smoking status group is shown in Table 3. Groups A and C had a higher percentage of male patients compared to female patients (67.5% and 61.9% males, respectively) (*p* = 0.004). The majority of patients in Group C underwent transplant using MUDs at 59% (overall *p*-value = 0.02). Group C also had a higher percentage of dual T-cell depletion for GVHD prophylaxis using PTCy combined with ATG, at 76.2%; however, this was borderline significant (overall *p*-value = 0.07). Finally, Groups A and B (both smokers and non-smokers, respectively, with pulmonary comorbidity) had a higher percentage of patients with HCT-CI score of ≥3, at 77.5% and 60.6% (*p* < 0.001), as well as a higher percentage of high-risk DRI at 27.5% and 23.9%, respectively (*p* < 0.001).

### 3.2. Hospital Admission Time

**Figure 1:**
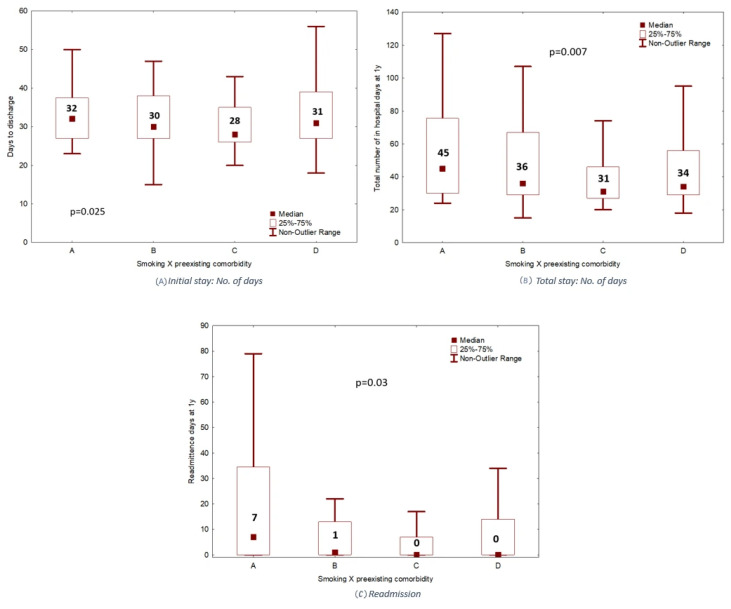
(**A**–**C**) Number of initial inpatient days, total inpatient days, and readmission days for Groups A, B, C, and D.

The median number of initial hospitalization days for the purpose of Allo-HCT was slightly higher in the smokers with preexisting pulmonary comorbidity (Group A) at 32 days, compared to Groups B, C, and D with 30, 28, and 31 days, respectively (*p* = 0.025) (Figure 1A). The median number of total days in hospital during the first year post-transplant (including for all readmissions for any reason) was 45 days for Group A, compared to 36, 31, and 34 days for Groups B, C, and D, respectively (*p* = 0.007) (Figure 1B), while the median number of actual readmission days during the first year was also significantly higher for Group A compared to the other Groups (*p* = 0.03) (Figure 1C). Group A also demonstrated a higher rate of ICU admissions (within one year following Allo-HCT) at 25% of patients, compared to 14.1%, 11.4%, and 11.6% for Groups B, C and D. However, there was no significant difference in the number of ICU admissions across the four groups (*p* = 0.2).

### 3.3. Post-Transplant Outcomes

**Figure 2:**
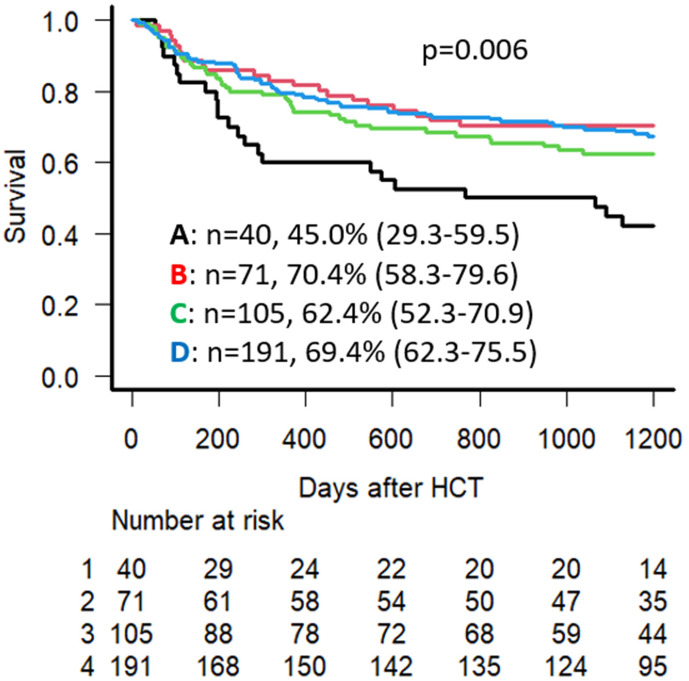
Univariate analysis for OS for Groups A, B, C, and D (survival % noted at 3 years following Allo-HCT) alongside the “Numbers at risk” data for each group.

**Figure 3:**
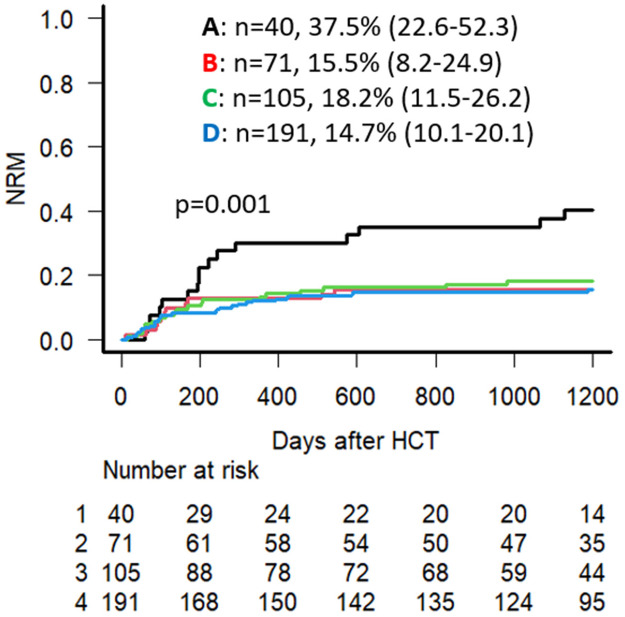
Univariate analysis for NRM for Groups A, B, C, and D (NRM % noted at 3 years following Allo-HCT) alongside the “Numbers at risk” data for each group.

Univariate analysis (UVA) demonstrated that the 3-year OS for the entire cohort was 65.3% (95% CI: 60.5–69.8), with an overall 3-year NRM of 18.0% (95% CI: 14.4–21.9). The 3-year OS is lowest in Group A at 45.0% (95% CI: 29.3–59.5), compared to 70.4% (95% CI: 58.3–79.6) in Group B, 62.4% (95% CI: 52.3–70.9) in Group C, and 69.4% (95% CI: 62.3–75.5) in Group D (*p* = 0.006) (Figure 2). Group A had the highest NRM at 37.5% (95% CI: 22.6–52.3) compared to 15.5% (95% CI: 8.2–24.9), 18.2% (95% CI: 11.5–26.2), 14.7% (95% CI: 10.1–20.1) in Groups B, C, and D, respectively (*p* = 0.001) (Figure 3). No significant difference was seen regarding CIR, with 20.5%, 23.9%, 28.8%, and 23.5% at 3 years post-transplant for Groups A, B, C, and D, respectively (*p* = 0.48).

**Figure 4:**
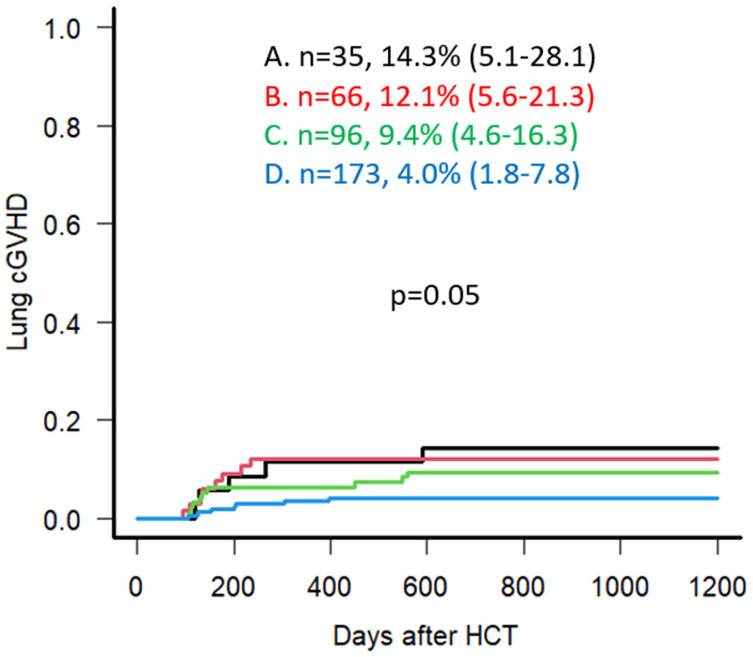
Univariate Analysis for Lung cGVHD incidence for Groups A, B, C, and D (incidence % noted at 3 years following Allo-HCT).

Regarding grade III-IV acute GVHD at 100 days post-transplant, Group A showed a higher but not significantly different frequency at 12.5% (95% CI: 4.5–24.8), while Groups B, C, and D demonstrated 9.9% (95% CI: 4.3–18.2), 6.7% (95% CI: 2.9–12.5), and 7.3% (95% CI: 4.2–11.6), respectively (*p* = 0.55). For moderate to severe chronic GVHD within the first two years post-transplant, no significant differences were seen, with 22.9% (95% CI: 10.5–38.0), 28.8% (95% CI: 18.4–40.1), 26.2% (95% CI: 17.7–35.3), and 20.8% (95% CI: 15.1–27.2) for Groups A, B, C, and D, respectively (*p* = 0.61). Interestingly, when specifically examining the incidence of pulmonary chronic GVHD, Group A demonstrated the highest proportion with 45% of total chronic GVHD cases with pulmonary involvement compared to 31%, 26%, and 10% for Groups B, C, and D, respectively (*p* = 0.01). Group A also demonstrated the highest proportion, with 14.3% of the group developing pulmonary chronic GVHD compared to 12.1%, 9.4%, and 4.0% for Groups B, C, and D, respectively (*p* = 0.05) (Figure 4).

### 3.4. Impact of Smoking Duration

**Figure 5:**
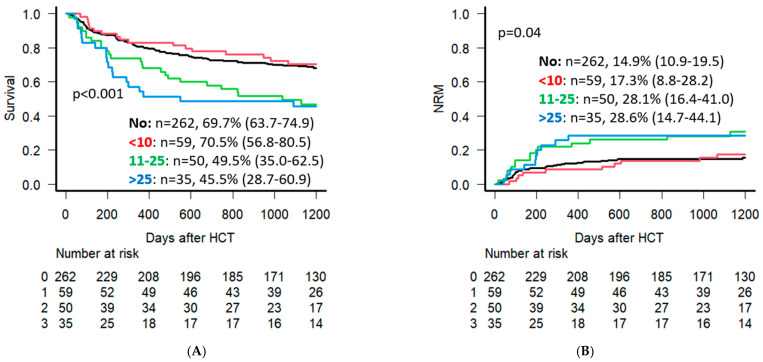
Univariate analysis for OS (**A**) and NRM (**B**) for patients with no smoking, <10, 11–25, and >25 pack-year smoking history (outcome % noted at 3 years following Allo-HCT) alongside the “Numbers at risk” data for each group.

In our UVA, patients with >25 pack-years smoking history had the lowest 3-year OS at 45.5% (95% CI: 28.7–60.9), compared to 49.5% (95% CI: 35.0–62.5) for 11–25 pack-years and 70.5% (95% CI: 56.8–80.5) for <10 pack-years (*p* < 0.001) (Figure 5A). Patients with <10 pack-years also had a lower 3-year NRM at 17.3% (95% CI: 8.8–28.2) compared to 11–25 pack years and >25 pack years with 28.1% (95% CI: 16.4–41.0) and 28.6% (95% CI: 14.7–44.1), respectively (*p* = 0.04) (Figure 5B).

When dividing smokers into Recent, Former, and Remote smoking history in UVA, Remote smokers had an improved 3-year OS (63.8%, 95% CI: 52.3–73.3) compared to Former (52.3%, 95% CI: 36.7–65.7) and recently discontinued smokers (43.3%, 95% CI: 21.1–63.7), although this difference was not statistically significant in our cohort (*p* = 0.29). For 3-year NRM, UVA also showed no significant difference: Remote smokers scored 23.5% (95% CI: 14.9–33.3), Former smokers 20.5% (95% CI: 10–33.5), and Recent smokers 30.5% (95% CI: 11.9–51.6) (*p* = 0.87).

### 3.5. Multivariable Analysis

In the multivariate analysis (MVA) for OS, HCT-CI ≥ 3 (HR = 1.54, 95% CI: 1.10–2.15, *p* = 0.01), high-risk DRI (HR = 2.16, 95% CI: 1.45–3.21, *p* < 0.001) and over 10 pack-years of smoking history (HR = 1.82, 95% CI: 1.15–2.88, *p* = 0.01) were independently associated with increased mortality. In the MVA for NRM, older age at Allo-HCT (HR = 1.03, 95% CI: = 1.01–1.04, *p* = 0.004), HLA mismatched unrelated donor use (HR = 1.78, 95% CI: = 1.12–2.83, *p* = 0.02), and smoking history with preexisting pulmonary comorbidity (Group A) (HR = 3.19, 95% CI: 1.75–5.82, *p* < 0.001) were all independently associated with an increased NRM (Table 4).

## 4. Discussion

In the presented study, we report on the impact of smoking history on Allo-HCT outcomes in a single center cohort. We demonstrated a significantly worse OS and NRM for the smoking history group with pulmonary comorbidity, Group A, and to a lesser extent the smoking history group without pulmonary comorbidity, Group C, compared to the other designated non-smoking groups. These findings were in concordance with previously reported findings in the literature, and point to a significant impairment in outcome related to smoking history combined with pulmonary comorbidity as determined on pre-transplant assessment. These findings may influence discussions with Allo-HCT candidates that have a similar history and comorbidity profile to the patients in the present study, especially regarding smoking cessation pre-transplant and post-transplant follow-up. Moreover, our study demonstrated that the number of inpatient hospital days for transplant for Group A was higher than that of any of the other designated groups, while Group A also had the highest number of readmission days within the first year following Allo-HCT. Therefore, besides the impact on survival outcomes, smoking history may also indirectly impact other healthcare-related parameters such as hospitalization costs and inpatient bed availability.

Regarding GVHD, the presented study did not show any significant impact on the overall incidence of acute and chronic GVHD. Upon dissecting this further, however, Group A showed a statistically significant trend towards increased chronic pulmonary GVHD at the rate of 45% of all chronic GVHD patients in that group. Bearing in mind the limitation of the relatively small number of patients involved in this subgroup, one can theorize that the synergistic effect of smoking and lung comorbidity (which in itself may be related to smoking) may contribute to the increased incidence of pulmonary GVHD in these patients.

When our smoking patient cohort was divided based on cumulative smoking dose and the timing of smoking history as described in the Methods Section, we demonstrated a significant negative impact of a smoking burden of >25 pack-years on OS compared to the other groups. This finding is also supported in the literature. Smoking more recently before Allo-HCT seemed to be associated with worse outcomes; however, this finding lacked statistical significance, probably due to small patient numbers. Recent smokers (having quit within the last year before transplant) showed the lowest 3-year OS at 43.3% and the highest NRM at 30.5%. Our findings support smoking cessation for Allo-HCT candidates as soon as possible before transplant.

### Smoking and Outcome Discrepancies Following Allo-HCT

Over the past few decades, multiple studies have been conducted to assess the effect of smoking on Allo-HCT. These studies did show conformity for the most part, but demonstrated a degree of discrepancy in their reported results.

Marks et al. [10] demonstrated in their cohort that the 5-year OS was lower in high-dose smokers compared to low-dose and nonsmokers, with survival rates of 50%, 62%, and 68%, respectively. NRM showed a similar increase in high-dose smokers at 50%, compared to 32% and 28% in low-dose and nonsmokers. The multivariate analysis showed the relative risk (RR) of relapse to be higher in any smokers compared to nonsmokers with a RR of 1.67, but no clear association with the high-dose group. These findings were significant only in the matched sibling donor group of patients but not in the unrelated donor group. No correlation was found between smoking and overall incidence of acute or chronic GVHD, similar to the findings in the presented study.

In another related study, Ehlers et al. [11] divided smokers into current smokers (within 1 year of Allo-HCT), former smokers (quitting prior to 1 y of Allo-HCT), and nonsmokers. The current smoking group was found to be associated with a worse OS (HR 1.88 v. 1.31) and a longer duration of initial hospital stay (46 v. 30 v. 26 days) compared to former and nonsmokers. Tran et al. [27] found an association between smoking and early respiratory failure (HR 1.33). They also demonstrated a trend for increased risk of disease relapse, but this was not statistically significant. Moreover, they did not show a clear association between smoking and NRM.

Savani et al. [12] showed that smoking increases the risk of pulmonary transplant-related mortality (PTRM), with a RR of 5.0 for patients that regularly smoked within 2 months prior to Allo-HCT and for a minimum of 2 years. In another study, Hanajiri et al. [28], divided smokers based on the Brinkman Index (BI) into low (BI < 400) and high dose (BI ≥ 400). High-dose smokers had a higher incidence of pulmonary complications at 46%, compared to 36% and 26% in low-dose and nonsmokers. However, OS, NRM and cumulative incidence of relapse at 3 years were all similar between the groups. Finally, Chang et al. [29] demonstrated that a history of smoking prior to Allo-HCT was found to be associated with an increased risk of disease relapse, with each pack-year history of smoking increasing the risk 1.7%. Meanwhile, no clear association between smoking and survival or mortality was identified in that study.

One study in particular has recently shown a link between smoking and the incidence of chronic GVHD [30]. Ohashi et al. divided smokers based on the Brinkman Index into nonsmokers, low (BI < 500) and high dose (BI ≥ 500) smokers. The high-dose group correlated to a higher risk of chronic GVHD at 57% v. 49%. Of note, the OS and disease free survival (DFS) were lower in the univariate analysis for high-dose smokers at 39% versus 52% OS, and 37% versus 47% DFS. Meanwhile, NRM, relapse incidence, and acute GVHD did not differ significantly. They showed that lung GVHD was not specifically correlated to the high-dose group. The study group speculated that the recency of smoking might be a factor increasing the incidence of chronic GVHD overall.

The outcome discrepancies in the above-described studies lead us to the hypothesis that different study design, sample sizes, transplant practices, or outcome definitions may be having an impact on outcome interpretation. There may also be an unidentified confounding variable influencing outcomes that has not been accounted for. The variables identified in our paper could be essential in explaining these findings moving forward.

## 5. Conclusions

Taking the results of our study into perspective, our results indicate that pulmonary chronic GVHD seems to be an important complication for smokers, possibly due to chronic tissue damage and negative immunomodulation on the cellular level. This seems to be observed more in smokers with concurrent pulmonary comorbidity. Additional studies investigating the impact of smoking history on individual acute and chronic GVHD subtypes may be of benefit. Other studies investigating the mechanisms behind these findings, such as altered lung microbiome or smoking-induced tissue damage, would provide further valuable insight.

While informative, our study demonstrates a number of limitations. Our study was performed at a single center with a limited sample size. Increasing the number of patients and possibly involving other centers across multiple locations with different transplant approaches may further consolidate our findings. Of note, while our analysis showed a trend towards worse outcomes with Recent smoking (although not statistically significant), it is important to note the small sample size in that group (n = 20). Our study was also retrospective in nature, and despite meticulous data collection, remains at risk for retrospective bias, as well as the possibility of missing data, particularly in regard to the documented details related to smoking history. A prospectively designed study involving multiple centers would mitigate these shortcomings. Another limitation is the relatively short follow-up duration for chronic GVHD assessment.

In conclusion, we demonstrated the negative synergistic effect of smoking and pre-transplant pulmonary comorbidities on survival outcomes as well as an increased incidence of pulmonary chronic GVHD. Our results may improve the prognostication of Allo-HCT outcomes for individuals with a significant smoking history and underlying lung comorbidities prior to transplant. Our findings underscore the importance of developing interventions with the goal of optimizing lung status prior to transplant, and the importance of smoking cessation as early as possible.

## Figures and Tables

**Table 1:** Patient- and transplant-related baseline characteristics.

Characteristics	N (%) or Median (Range)
N	407
Age	58 (18–76)
Sex (Male/Female)	194/213
Female donor/Male recipient	69 (17.0)
Diagnosis:	
AcL	259 (63.6)
ChL	14 (3.4)
MDS/MPN	92 (22.6)
Lymphoma	22 (5.4)
Non-malignancy	20 (4.9)
Source (BM/PBSC)	19/388
Frozen graft	92 (22.6)
CD34 dose	7.1 (0.3–19.4)
Donor:	
MRD	94 (23.1)
MUD	195 (47.9)
Haplo	79 (19.4)
MM URD	39 (9.6)
Donor age	30 (17–70)
RIC/MAC	246/161
GVHD prophylaxis:	
ATG	81 (19.9)
Other	8 (2.0)
PTCy	54 (13.3)
PTCy + ATG	264 (64.9)
ATG dose:	
no	62 (15.2)
2 mg/kg	255 (62.7)
4.5 mg/kg	90 (22.1)
KPS < 90	67 (16.5)
HCT-CI ≥ 3	130 (31.9)
DRI High	58 (14.3)
Follow-up (months)	47.6 (17.1–64.4)

Abbreviations: AcL, Acute Leukemia; ATG, Anti-thymocyte Globulin; BM, Bone Marrow; ChL, Chronic Leukemia; DRI, Disease Risk Index; GVHD, Graft-vs-Host Disease; Haplo, haploidentical; HCT-CI, HCT-Comorbidity Index; KPS, Karnofsky Performance Status; MAC, Myeloablative Conditioning; MDS, Myelodysplastic Syndrome; MPN, Myeloproliferative Neoplasm; MRD, Matched Related Donor; MM URD, Mismatched Unrelated Donor; MUD, Matched Unrelated Donor; PBSC, Peripheral Blood Stem Cells; PTCy, Post-transplant Cyclophosphamide; RIC, Reduced-Intensity Conditioning.

**Table 2:** Group distribution based on pulmonary comorbidity and pre-transplant smoking status.

Characteristics	N (%)	<10 Packs/Year	11–25 Packs/Year	>25 Packs/Year
**Smoking information:**				
**A**. Smokers with preexisting pulmonary comorbidity	40 (9.8)	16 (40.0)	11 (27.5)	13 (32.5)
**B**. Non-smokers with preexisting pulmonary comorbidity	71 (17.4)	0	0	0
**C**. Smokers with no preexisting pulmonary comorbidity	105 (25.8) (one patient with missing data)	43 (41.0)	39 (37.1)	22 (21.0)
**D**. Non-smokers with no preexisting pulmonary comorbidity	191 (46.9)	0	0	0
**Cigarette packs/year**				
Non-smokers	262 (64.5)			
<10	59 (14.5)			
11–25	50 (12.3)			
>25	35 (8.6)			

**Table 3:** Patient and transplant characteristics distributed by pulmonary comorbidity/smoking status group.

Characteristics	Group A	Group B	Group C	Group D	*p*-Value
N	40	71	105	191	
Age	57 (19–72)	51 (19–71)	59 (20–74)	58 (18–76)	<0.001
Male Sex	27 (67.5)	28 (39.4)	65 (61.9)	93 (48.7)	0.004
Frozen graft	10 (25.0)	16 (22.5)	27 (25.7)	39 (20.4)	0.75
Donor:					0.02
MRD	11 (27.5)	19 (26.8)	16 (15.2)	48 (25.1)	
MUD	19 (47.5)	29 (40.8)	62 (59.0)	85 (44.5)	
Haplo	10 (25.0)	16 (22.5)	13 (12.4)	40 (20.9)	
MM URD	0	7 (9.9)	14 (13.3)	18 (9.4)	
RIC	26 (65.0)	44 (62.0)	61 (58.1)	115 (60.2)	0.88
GVHD prophylaxis:					0.07
ATG	11 (27.5)	17 (23.9)	18 (17.1)	35 (18.3)	
Other	2 (5.0)	2 (2.8)	0	4 (2.1)	
PTCy	3 (7.5)	11 (15.5)	7 (6.7)	33 (17.3)	
PTCy + ATG	24 (60.0)	41 (57.7)	80 (76.2)	119 (62.3)	
KPS < 90	7 (17.5)	17 (23.9)	14 (13.3)	29 (15.2)	0.28
HCT-CI ≥ 3	31 (77.5)	43 (60.6)	24 (22.9)	32 (16.8)	<0.001
DRI High	11 (27.5)	17 (23.9)	8 (7.6)	22 (11.5)	<0.001
>25 packs/year	13 (32.5)	0	22 (21.0)	0	

Abbreviations: ATG, Anti-thymocyte Globulin; DRI, Disease Risk Index; GVHD, Graft-vs-Host Disease; Haplo, haploidentical; HCT-CI, HCT-Comorbidity Index; KPS, Karnofsky Performance Status; MRD, Matched Related Donor; MM URD, Mismatched Unrelated Donor; MUD, Matched Unrelated Donor; PTCy, Post-transplant Cyclophosphamide; RIC, Reduced-Intensity Conditioning.

**Table 4:** Multivariable analysis for OS and NRM.

	*p*-Value	Hazard Ratio	95% Hazard Ratio Lower CL	95% Hazard Ratio Upper CL
MVA For OS				
Age	0.063	1.012	0.999	1.024
High DRI	0.0002	2.157	1.449	3.210
HCT-CI ≥ 3	0.013	1.536	1.096	2.153
<10 Pack-years	0.805	0.939	0.569	1.548
11–25 Pack-years	0.0105	1.819	1.150	2.879
>25 Pack-years	0.063	1.624	0.974	2.706
MVA For NRM				
Age	0.004	1.026	1.008	1.045
HLA Mismatch	0.015	1.783	1.119	2.839
Group A	0.0001	3.194	1.752	5.821
Group B	0.641	1.182	0.586	2.383
Group C	0.509	1.216	0.681	2.172
Group D	-	-	-	-

Abbreviations: DRI, Disease Risk Index; HCT-CI, HCT-Comorbidity Index; HLA, human leukocyte antigen, MVA, multivariable analysis; NRM, non-relapse mortality; OS, overall survival.

## Data Availability

Data is unavailable due to privacy or ethical restrictions.
